# Advances and challenges in experimental models for Epstein–Barr virus research

**DOI:** 10.1002/mlf2.70081

**Published:** 2026-08-11

**Authors:** Gulimire Wufuer, Jiabao Tang, Tingdong Li, Shengxiang Ge, Jun Zhang, Ningshao Xia

**Affiliations:** ^1^ State Key Laboratory of Vaccines for Infectious Diseases, Xiang An Biomedicine Laboratory, Department of Laboratory Medicine, School of Public Health Xiamen University Xiamen China; ^2^ National Institute of Diagnostics and Vaccine Development in Infectious Diseases, Collaborative Innovation Center of Biologic Products, National Innovation Platform for Industry‐Education Integration in Vaccine Research, NMPA Key Laboratory for Research and Evaluation of Infectious Disease Diagnostic Technology Xiamen University Xiamen China

**Keywords:** animal models, Epstein–Barr virus (EBV), viral pathogenesis, 2D cell lines, 3D organoids

## Abstract

Epstein–Barr virus (EBV), the first identified human oncovirus, is a ubiquitous γ‐herpesvirus etiologically linked to diverse malignancies, lymphoproliferative disorders, and autoimmune diseases. Experimental models are pivotal for unraveling the infection and pathogenesis mechanisms, life cycle regulation, as well as development and evaluation of antiviral vaccines and therapeutics. Traditional two‐dimensional (2D) cell lines (e.g., lymphoblastoid cell lines [LCLs] and NPC43) have provided foundational insights into viral latency and oncogenesis, but lack physiological complexity. Emerging three‐dimensional (3D) models, such as air–liquid interface (ALI) cultures, spheroids, and patient‐derived organoids (PDOs), better mimic tissue architecture and tumor microenvironments and yet face challenges in scalability, dynamic microenvironments, and immune component integration. Animal models, such as humanized mice, rabbits, and tree shrews, have been developed that overcome *in vitro* limitations by recapitulating systemic interactions, immune responses, and disease progression. Despite these advances, persistent challenges remain, including species‐specific constraints, incomplete modeling of the tumor microenvironment, partial immune system representation, and limited disease heterogeneity. This review systematically summarizes the technical features, applicability, and limitations of current models, provides guidance for selecting experimental models in further studies, and emphasizes the need for innovative, patient‐tailored platforms to unravel EBV's lifelong pathogenesis and facilitate the translation of EBV vaccines and therapeutics.

## INTRODUCTION

The Epstein–Barr virus (EBV), a γ‐herpesvirus with a 172‐kb double‐stranded DNA genome^1^, infects over 90% of the population globally and establishes lifelong latency in B lymphocytes after primary infection^2^. While most EBV infections remain asymptomatic, the virus is etiologically linked to diverse diseases, including infectious mononucleosis (IM), lymphoproliferative disorders, autoimmune diseases, and malignancies such as nasopharyngeal carcinoma (NPC), gastric cancer (GC), and Burkitt lymphoma (BL)^3^, ^4^. Collectively, EBV infection causes over 200,000 cancer cases annually^5^, ^6^.

EBV transmission primarily occurs via saliva^7^, and involves a biphasic life cycle characterized by latent infection and lytic reactivation to achieve lifelong persistence in human hosts^8^. In latency, the viral genome persists as episomes and replicates synchronously with host cell division, ensuring vertical transmission to daughter cells^8^. Latency is maintained through the expression of a limited set of viral genes, including Epstein–Barr nuclear antigens (EBNAs) and latent membrane proteins (LMPs), as well as non‐coding RNAs (e.g., EBERs [Epstein–Barr virus‐encoded small RNAs] and miRNAs), which collectively suppress lytic reactivation and modulate host signaling pathways to promote cell survival and immune evasion^9^.

Reactivation of the latent virus into lytic replication may occur *in vivo*, which is essential for viral shedding, dissemination, and pathogenesis^10^. This process is tightly regulated through a cascade of transcriptional activation that is initiated by the master transcriptional activators BZLF1 (Zta) and BRLF1 (Rta)^11^, ^12^. During the lytic phase, the viral genome is amplified ~100‐fold via a rolling‐circle mechanism at the lytic origin (oriLyt), leveraging both the viral and host replication machinery^8^, ^13^. Subsequently, structural proteins are synthesized, ensuring packaging of progeny genomes into preassembled capsids. Virion maturation involves sequential envelopment at the nuclear membranes and cytoplasmic membranes, which ultimately leads to the release of mature progeny virions^14^. Prolonged latent infection and intermittent reactivation can lead to the development of various diseases^15^. However, dynamic switching of EBV cycles between latent and lytic states, molecular mechanisms underlying immune evasion during latency, and how EBV drives malignant transformation remain incompletely understood^7^. Addressing these issues requires robust experimental models that recapitulate EBV's complex life cycle and host interactions.

EBV pathogenic studies mainly include cell models *in vitro* and animal models *in vivo*, which have been widely applied in the research of EBV infection mechanisms, drug and vaccine evaluation, and pathogenic mechanisms of EBV‐associated diseases. The development of CRISPR/Cas9, organoid models, cell‐ or patient‐derived xenografts, and humanized mice has significantly accelerated research progress. EBV infection models have evolved from simplistic cell lines to sophisticated humanized systems; yet, gaps remain in mimicking immune interactions and tumor progression. This review summarizes the advances in cell and animal models for EBV infection and pathogenesis, highlighting their transformative role in elucidating pathogenesis and evaluating targeted therapies. These advances provide direction for the establishment of novel experimental models and the evaluation of drugs and vaccines in the future.

## *IN VITRO* MODELS FOR EBV INFECTION AND PATHOGENESIS

EBV experimental models *in vitro* provide critical platforms for dissecting viral life cycle, pathogenesis, and tumor microenvironment (TME) dynamics. Currently, *in vitro* experimental models for EBV commonly include two‐dimensional (2D) cell line and three‐dimensional (3D) organoid culture models. The 2D models enable high‐throughput mechanistic studies, while 3D organoid models, derived from patient tissues or stem cells, further recapitulate the structural and functional complexity of native tissues, allowing co‐culture with stromal or immune cells to mimic tumor‐immune crosstalk and microenvironmental influences^16^.

### 2D cell line models

*In vivo*, EBV shows tropism for B lymphocytes and epithelial cells. Infection of B cells *in vitro* leads to their immortalization, generating lymphoblastoid cell lines (LCLs)^17^. LCLs retain viral episomes and mimic *in vivo* EBV‐driven B cell proliferation, enabling investigations into latency programs and immune evasion modulation of EBV^18^, ^19^, ^20^. To date, multiple EBV‐positive cell lines have been successfully isolated from patient tumor tissues, and these cell lines are invaluable for elucidating the life cycle of EBV and pathogenesis of EBV‐associated malignancies. Examples encompass C666‐1, NPC43, and C17 derived from NPC^21^, ^22^, ^23^, SNU719 and YCCEL1 isolated from gastric carcinoma patients^24^, ^25^, and Mutu I cells derived from BL patients.

In EBV‐positive cell lines, EBV exists in a latent phase that is tightly regulated by epigenetic and cellular signaling cues, and the gene expression of EBV adheres to a stringent and specific pattern within various cell lines, including EBV‐positive BL cell lines, NPC cell lines, and LCLs. These distinct cell lines are characterized by their respective encoding of latency programs I, II, and III, each of which dictates a restricted expression profile of specific viral genes. For instance, the latency I program is characterized by the restricted expression of EBNA1, EBERs, and viral *Bam*HI‐A rightward transcripts (BARTs)^26^. In contrast, the latency II program is marked by the expression of EBNA1, LMPs, EBERs, and associated transcripts^26^. Latency III is defined by the expression of a full complement of EBNA proteins, LMPs, EBERs, EBV‐encoded microRNAs, and BART transcripts^26^. Ectopic expression of the Zta protein potently induces lytic reactivation, thereby providing a robust platform to dissect the mechanisms governing the viral latency‐to‐lytic transition. In addition, the EBV‐positive cell lines retain native viral gene expression patterns and tumor‐specific mutations, offering insights into transcriptional profiles consistent with *in vivo* observations^27^, thereby offering biologically relevant platforms for pathobiological studies underlying EBV‐associated malignancies. Meanwhile, advancements further leverage bacterial artificial chromosome (BAC)‐engineered EBV strains, which incorporate fluorescent reporter genes to enable precise tracking, genetic manipulation, and establishment of isogenic cell lines^28^. Such models are indispensable for delineating EBV's role in tumor initiation, progression, and metastasis, as well as for mapping infection‐associated genomic alterations, signaling dysregulation, and host–pathogen interactions.

Additionally, various EBV‐negative cell lines are instrumental in elucidating the early events of EBV infection, cellular transformation processes, and the mechanisms of EBV infection and transformation within epithelial cells. For instance, the HK1 cell line, a well‐differentiated NPC cell line that lost EBV genomes during passage culture^29^, serves as a valuable model for investigating the biological characteristics of NPC and for studying EBV pathogenesis in nasal epithelial cells^30^, ^31^, ^32^, ^33^. The Akata cell line (derived from BL), while inherently EBV‐negative, can be experimentally infected with EBV to establish a robust infection model *in vitro*, thereby facilitating the study of EBV infection mechanisms and the viral life cycle^34^. Furthermore, the human nasopharyngeal epithelial cell lines NP69 (immortalized by the SV40 large T antigen)^35^ and NP460hTert (immortalized by telomerase)^36^, provide a physiologically relevant context for examining EBV infection and transformation mechanisms within epithelial cells.

These EBV infection‐related cell lines collectively enhance our understanding of the initial stages of EBV infection, the processes of cellular transformation, and the specific mechanisms by which EBV interacts with and alters infected cells, contributing to the broader comprehension of EBV pathogenesis and its role in carcinogenesis. The development and utilization of these cell lines not only advance our comprehension of EBV's biological characteristics but also furnish an essential experimental foundation for the diagnosis, treatment, and prevention of EBV‐associated diseases.

Despite significant progress in EBV research, *in vitro* modeling of epithelial cell infection remains technically challenging due to several limitations. First, EBV shows low infection efficiency in monolayer epithelial cultures. Strategies like co‐culture of B cells and epithelial cells to mediate viral transfer or receptor overexpression (e.g., integrins) partially address this but fail to sustain viral episomes long‐term^37^, ^38^. This instability complicates mechanistic studies of latent and lytic viral programs in epithelial systems. Second, EBV replication *in vivo* is tightly linked to epithelial differentiation states. Terminally differentiated cells support robust viral replication and dissemination^14^, ^39^. However, conventional 2D cultures fail to recapitulate the polarized architecture and differentiation gradients critical for sustaining viral entry, persistence, and progeny release observed in physiological contexts^40^. Finally, the TME in EBV‐associated malignancies exerts profound influences on viral oncogenesis. Traditional 2D systems cannot model these multicellular interactions or biomechanical cues that are critical for EBV‐driven carcinogenesis.

### 3D culture models

Emerging 3D culture systems, including air–liquid interface (ALI) models, spheroids, and patient‐derived organoids (PDOs), are revolutionizing the study of EBV infection and oncogenesis by recapitulating the TME through 3D spatial organization, stromal integration, and immune cell inclusion^41^, ^42^. These models leverage diverse EBV‐associated epithelial cell lines, such as C666‐1 and HK1, thereby bridging critical gaps between traditional 2D cultures and *in vivo* complexity, and offering unprecedented insights into viral–host dynamics.

**Figure 1:**
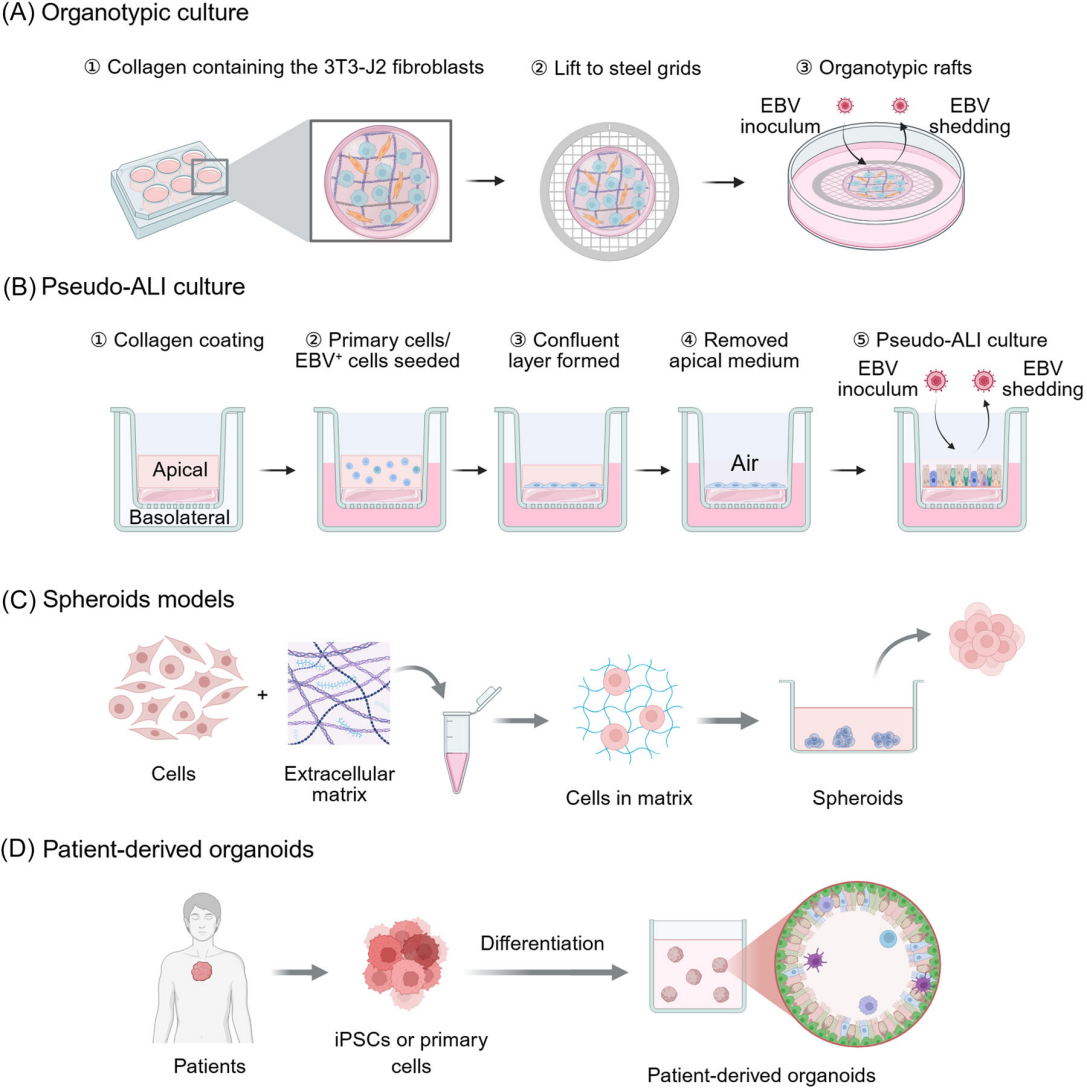
Construction processes of different 3D culture systems. (A) Organotypic culture. Cells are seeded onto a collagen matrix embedded with supporting 3T3‐J2 fibroblasts. This construct is lifted onto steel grids to establish an air–liquid interface, creating stratified “rafts” suitable for apical EBV inoculation. (B) Pseudo‐ALI (air–liquid interface) culture. Primary or EBV‐positive cells are seeded onto collagen‐coated permeable membrane inserts. Upon reaching confluence, the apical medium is removed to expose the cells to air, facilitating polarization and apical viral shedding. (C) Spheroid models. Single cells are suspended within an extracellular matrix (ECM) hydrogel and plated, where they self‐assemble into three‐dimensional multicellular aggregates. (D) Patient‐derived organoids. Primary cells or iPSCs are isolated from patient tissue and subjected to directed differentiation protocols to generate complex, self‐organizing 3D structures that mimic native tissue architecture. EBV, Epstein–Barr virus; iPSCs, induced pluripotent stem cells. This figure was created by Bio‐Render. Wufuer, G. (2026) https://www.biorender.com.

The ALI system is a widely used experimental model in cell biology and tissue engineering. It replicates the *in vivo* growth environment of cells at the ALI by exposing them to both air and liquid. This dual exposure enables the system to accurately reproduce the natural polarity and stratified structure of epithelial tissues^43^, thereby providing a more physiologically relevant context. Among ALI models, organotypic raft cultures represent a specialized form that produces stratified squamous epithelium, typically utilizing primary or immortalized cells^30^, ^44^. These cultures have been particularly valuable in EBV studies, as they mimic the stratified oral epithelium where cellular differentiation triggers EBV lytic infection^45^, ^46^. In contrast, nasopharyngeal “pseudo‐ALI” cultures, which can utilize conditionally reprogrammed cells, recapitulate the distinctive pseudostratified epithelium of the nasopharynx, particularly the lymphoid‐rich fossa of Rosenmüller, a predominant site of NPC tumorigenesis^43^. This model can support both latent and lytic EBV infection in a donor‐dependent manner, which may contribute to viral persistence and shedding in the nasopharynx^43^. Both organotypic raft cultures and nasopharyngeal pseudo‐ALI cultures are tailored to replicate specific *in vivo* epithelial structures relevant to EBV biology. For organotypic raft cultures (Figure 1A), cells are first embedded in collagen‐containing 3T3‐J2 fibroblasts to mimic the stromal microenvironment^44^, ^45^. The construct is then lifted onto steel grids, allowing the formation of stratified squamous epithelium—a structure critical for modeling EBV lytic infection in the oral cavity, where cellular differentiation triggers viral reactivation^44^, ^45^. Nasopharyngeal pseudo‐ALI cultures (Figure 1B) begin with seeding primary or EBV‐positive cells onto a collagen‐coated, semi‐permeable membrane^43^. Initially, culture medium is supplied to both the apical and basolateral compartments to promote cell adhesion and proliferation until a confluent layer forms^43^. Subsequently, the apical medium is removed, creating an ALI that drives the development of a pseudostratified epithelium. Both models foster polarized, differentiated cellular structures that closely resemble *in vivo* tissues, enhancing the reliability of experiments investigating EBV pathogenesis in anatomically relevant contexts^43^. Despite its physiological relevance, the ALI model faces limitations, including prolonged establishment timelines and restricted scalability. Compared to conventional 2D cell culture models, the establishment of the ALI model necessitates a prolonged time frame, with the establishment and maturation processes varying depending on the cell type. While ALI cell cultures derived from primary cells typically require 2–4 weeks of cultivation, cell lines may complete the process in as soon as 1 week. It should be noted that the former show physiological and pathological characteristics more similar to those found in *in vivo* tissues^47^. This extended period is essential for cells to undergo differentiation, maturation, and the formation of 3D structures that closely mimic *in vivo* conditions, thereby recapitulating the physiological architecture and barrier function observed *in vivo* ^47^. To address these challenges, researchers have developed alternative 3D platforms, such as 3D spheroids and PDO models.

3D spheroids (Figure 1C) are primarily formed through intercellular adhesion, specifically cell aggregation, where the physical and chemical properties of their growth culture prompt cells to aggregate, forming 3D structures that replicate oxygen and nutrient gradients akin to *in vivo* TME, enabling the study of heterogeneous cell populations. 3D spheroids can be constructed from immortalized cell lines, primary cells, or fragments of human tissues. Spheroid formation begins within 24–48 h after seeding, and they typically reach a mature, compact state suitable for most experiments within 3–7 days. Siva Sankar et al. systematically outlined protocols for spheroid generation, emphasizing that the 3D spheroid model recapitulates the TME, thereby providing a scalable platform for high‐throughput drug screening and functional analysis of cancer hallmarks^48^. In contrast, organoids leverage the self‐organizing properties of stem cells, which can self‐renew and differentiate *in vitro* to generate 3D structures containing a variety of differentiated and functionally diverse cell lineages and also feature concentration gradients of oxygen and nutrients, effectively simulating the microenvironment of tumors or other tissues *in vivo* (Figure 1C).

PDO models (Figure 1D) utilize tissue samples from patients, thereby preserving the patients' genetic backgrounds and disease characteristics. Both spheroid and organoid models, when cultured *in vitro*, provide a more complex extracellular environment and intricate cell‐to‐cell and cell‐to‐matrix interaction networks than traditional 2D cultures. These models can self‐organize to form 3D structures that closely resemble the original tissue, encompassing various cell types and hierarchical levels, and partially retain the physiological functions and response characteristics of the original tissue *in vitro*. When constructing a 3D culture model, it is crucial to select appropriate cell lines based on the research objective, such as tumor cell lines and stem cell lines. The single‐cell suspension is then inoculated into a medium containing a matrix gel, such as Matrigel, to promote the self‐organization of cells into a 3D structure (Figure 1D). During this process, specific growth factors, hormones, or other supplements must be added to support cell growth and the formation of cell aggregates. Typically, organoids require 2–4 weeks or longer to fully mature and show the desired characteristics. Furthermore, through methods such as morphology, gene expression, and protein analysis, it can be verified whether the PDO model retains the characteristics of the original tissue.

While 3D models excel in TME mimicry, technical hurdles persist. Spheroids and organoids often suffer from incompatibility with high‐resolution imaging and flow cytometry due to their dense architecture^49^. Additionally, high costs, variable success rates, and stromal/immune component exclusion limit broader adoption. Nevertheless, these applications demonstrate transformative potential, particularly in drug discovery and in predicting how patients with hypoxic NPC will respond to radiotherapy^50^.

## ANIMAL MODELS OF EBV INFECTION AND PATHOGENESIS

While *in vitro* models are invaluable for basic research and drug development, they have inherent limitations. These models often fail to replicate the dynamic microenvironments and organotypic interaction characteristic of living organisms. Simulation of holistic physiological responses, including the intricate regulation of the immune system, metabolic processes, and inter‐organ crosstalk, presents a significant challenge. Consequently, although cell models offer valuable insights, their capacity to fully emulate the comprehensive functionality of organisms and the multifaceted progression of diseases is limited. To address these limitations, animal models play a pivotal role in biomedical research. They provide an experimental milieu that more closely recapitulates human physiological and pathological processes, serving as a crucial bridge between cell models and human studies. Animal models enable a more nuanced understanding of biological mechanisms and disease dynamics, ultimately contributing to the advancement of medical science and the development of therapeutic strategies.

**Figure 2:**
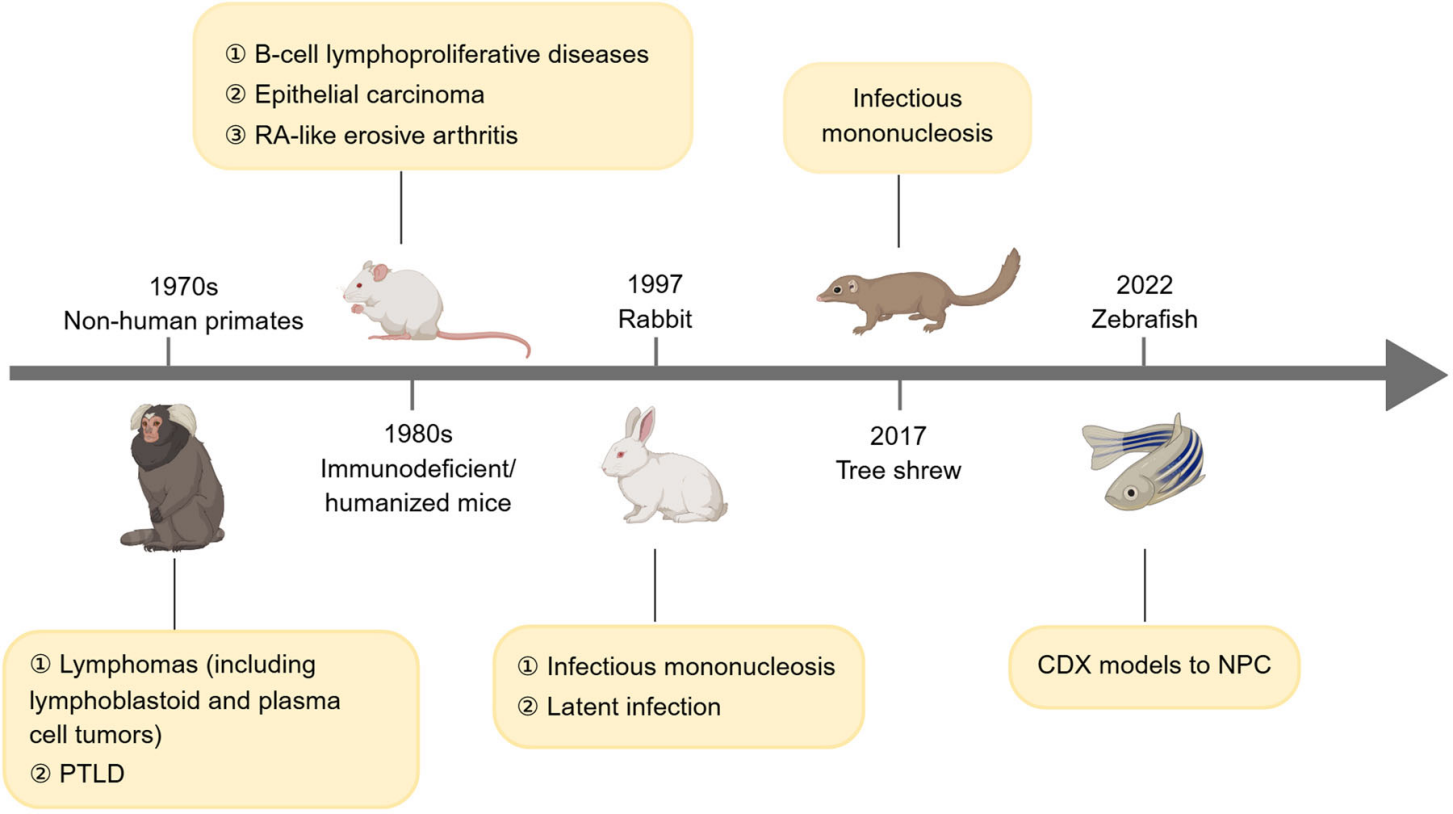
Application timelines and simulative disease spectra of diverse animal models. Disease types that the model can reproduce are outlined. CDX, cell‐derived xenograft; NPC, nasopharyngeal carcinoma; PTLD, post‐transplant lymphoproliferative disorder; RA, rheumatoid arthritis. This figure was created by Bio‐Render. Wufuer, G. (2026) https://www.biorender.com.

Naturally, EBV primarily infects humans, eliciting a range of specific biological responses and diseases. The virus's species specificity presents significant challenges in studying its infection mechanisms, pathogenicity, and immune evasion. Despite these hurdles, certain animal models, particularly genetically modified or humanized mice, can mimic some biological processes of EBV infection. Consequently, animal models remain indispensable for elucidating EBV pathogenesis, conducting preclinical drug screening, and validating therapeutics. These models, including non‐human primates (NHPs), rodents, rabbits, tree shrews, and zebrafish, recapitulate distinct aspects of EBV infection and oncogenesis, albeit with species‐specific limitations (Figure 2)^51^.

### NHP models

NHPs, particularly New World primates like cotton‐top tamarins (*Saguinus oedipus*), show key features that closely mirror humans, making them historically pivotal in EBV research. This model supports natural EBV‐like infection through oral transmission, recapitulating viral tropism for B cells and epithelial cells, as well as the establishment of lifelong latency—a hallmark of human EBV persistence. Early studies demonstrated that EBV inoculation in tamarins and cotton‐top marmosets induced B cell lymphomas, mirroring human post‐transplant lymphoproliferative disorder (PTLD)^52^, ^53^. However, ethical and conservation concerns have limited their use, since both species are critically endangered. Old World NHPs, especially rhesus macaques (*Macaca mulatta*), primarily due to their large population size and proven adaptability to laboratory breeding conditions, have become prevalent model organisms for studying human viral infections. Although the rhesus macaque model is permissive to lymphocryptovirus (rhLCV), which shows significant genomic conservation with EBV^54^, ^55^, it is refractory to stable EBV infection and B cell transformation^56^, posing limitations for direct EBV challenge studies^57^. Even so, rhesus macaques show symptoms upon EBV infection that closely resemble those observed in humans, including virus shedding in oral mucosa and potential transmission through close contact^58^. This model remains valuable for studying key aspects of EBV pathogenesis. These NHP models are particularly useful for EBV research because of their close evolutionary relationship with humans and conserved immune responses, enabling effective evaluation of neutralization, vaccine efficacy, and immune responses to EBV‐like lymphocryptoviruses^59^, ^60^.

### Rodent models

Mice naturally possess resistance to EBV, which complicates the study of EBV infection. Consequently, mouse models used to investigate EBV‐associated malignancies are often immunocompromised. Humanized mice, which have functional components of the human immune system engrafted into them, are susceptible to EBV targeting hematopoietic immune system cells and can mount cellular and humoral immune responses to the virus. Among the various humanized mouse models, those with human hematopoietic stem cells (Hu‐SRC/HSC) are commonly used in EBV challenge experiments. EBV can induce robust B cell transformation and proliferation in these models, mimicking aspects of EBV‐driven B cell acute lymphoblastic leukemia (B‐ALL)^61^ and B cell lymphoproliferative disorders (LPDs)^62^. However, other EBV‐associated malignancies, such as BL, Hodgkin's lymphoma, and NPC, remain to be characterized in these models.

Humanized mice are commonly used for the preliminary assessment of vaccine or neutralizing antibody efficacy during direct EBV challenges. However, the incomplete reconstitution of their immune system restricts a thorough assessment of prophylactic vaccines. Specifically, the engrafted human immune cells fail to fully recapitulate the complexities of the human immune response, as evidenced by an incomplete cellular composition, functional immaturity, and the absence of a physiological immune microenvironment. Additionally, the lack of key human epithelial cells, which are integral to EBV infection, further constrains the applicability of humanized mice in comprehensive EBV research endeavors. This limitation hinders a comprehensive evaluation of the prophylactic efficacy of vaccines against EBV infection, particularly in terms of mucosal immunity and the role of memory B cells in long‐term protection.

Research on EBV‐related malignancies predominantly relies on xenograft models to elucidate the virus's impact on tumorigenesis. These models provide a crucial *in vivo* platform for investigating tumor growth, metastatic potential, and therapeutic responses by engrafting human tumor cells or tissues into immunocompromised hosts^63^. Specifically, a cell‐derived xenograft (CDX) model that subcutaneously or orthotopically implants EBV‐positive tumor cells (e.g., C666‐1 and NPC43) into immunocompromised mice replicates NPC pathology^23^ and provides a valuable means for evaluating personalized treatment strategies, allowing researchers to more faithfully replicate individual patient tumor profiles and to tailor therapeutic regimens accordingly. Compared to the CDX model, the patient‐derived xenograft (PDX) model is constructed by transplanting tumor tissues from patients into immunodeficient mice. This approach better preserves the histological, morphological, and molecular characteristics of the original patient tumors, including gene mutations, expression profiles, and heterogeneity. The PDX model more closely mimics the biological characteristics of human tumors in terms of the TME, cancer stem cells (CSCs), and tumor heterogeneity. Consequently, it is more suitable for drug screening and personalized medical research^64^. However, the lengthy establishment time (4–8 months) and low success rates have limited their application in EBV‐related malignancies^65^. Notably, the current limited data show that detection of EBV infection in primary tumors may potentially alleviate the development of PDX lymphoma, suggesting a potential link between viral infection and the development of malignant tumors^64^, ^66^.

### Rabbit models

Early studies dismissed rabbits as refractory to EBV‐induced tumorigenesis despite transient increases in viral DNA and viral capsid antigen (VCA)‐IgG titers postinoculation^67^, ^68^. However, recent advances have revealed that immunosuppressed rabbits can sustain EBV replication with latency III gene expression^69^, mirroring the conditions found in immunocompromised human hosts. This paradigm shift is further supported by evidence that oral EBV transmission in rabbits recapitulates the natural route of primary infection in humans^70^. Consistent with this model, experimental data indicate that following natural infection via intranasal or oral routes, EBV‐DNA and/or mRNA remain detectable for several hundred days post‐viral inoculation, suggesting persistent viral replication and establishment of viral latency^70^, ^71^. The clinical significance of this persistent infection is evidenced by various pathologies in infected rabbits, including transient splenomegaly, brief infiltration of EBER1‐positive lymphocytes in hepatic biopsy specimens, elevated anti‐VCA immunoglobulin titers, increased EBV‐DNA load in peripheral blood, and systemic immune activation indicative of ongoing viral pathogenesis^69^, ^71^, ^72^. Rabbit models effectively recapitulate EBV‐associated lymphoproliferative disorders, hemophagocytic syndrome, and virus‐induced pathologies, demonstrating their utility for studying disease mechanisms and evaluating therapeutics^69^, ^73^. These models have proven particularly valuable for preclinical vaccine assessment, as shown by EBV virus‐like particle (VLP) immunogenicity studies and their ability to simulate reactivation under immunosuppression, despite limitations in achieving complete immune protection or depletion^69^, ^73^, ^74^, ^75^. Although the rabbit model has advantages, including low maintenance costs, ethical feasibility compared to NHPs, and compatibility with longitudinal studies of chronic infection, it fails to develop EBV‐associated malignancies (e.g., lymphomas or carcinomas) observed in humans or humanized mice, thereby restricting its utility in oncogenesis research.

### Tree shrew models

The tree shrew, a small mammal closely related to primates, has emerged as a compelling model for studying EBV infection and oncogenesis. Its genetic similarity to primates (~93% genome homology), compact size, and cost‐effective husbandry address ethical and logistical challenges of NHP models^76^, ^77^. Tree shrews naturally show susceptibility to EBV, supporting viral replication and latency‐associated gene expression (e.g., LMP1 and EBNA2), with pathological features resembling human EBV‐associated hepatitis and lymphoma‐like lesions^78^, ^79^. These traits enable studies on viral tropism, immune evasion, and chronic infection dynamics in immunocompetent hosts, providing a critical advantage over humanized mice requiring immunosuppression. Furthermore, tree shrews can recapitulate oral EBV transmission and spontaneous lytic reactivation, bridging gaps between *in vitro* systems and human clinical manifestations^79^. However, EBV‐induced malignancies in tree shrews remain less defined compared to human diseases (e.g., nasopharyngeal carcinoma), and while their immune system is functional, it diverges from humans in T cell receptor diversity and cytokine responses, complicating translational extrapolation^76^, ^77^.

### Others

The zebrafish, with its conserved genome, shares significant homology with humans, including key oncogenes and tumor suppressors, facilitating functional studies of EBV‐driven carcinogenesis^80^. The optical transparency of zebrafish embryos combined with advanced imaging technologies enables real‐time, *in vivo* visualization of tumor initiation, progression, and metastasis, providing unique insights into the spatiotemporal dynamics of oncogenesis that are unattainable through traditional *in vitro* models^81^, ^82^, ^83^, ^84^. Furthermore, the highly conserved immune system, encompassing T cells, B cells, and macrophages, facilitates the study of immune evasion strategies and host–pathogen interactions, while the transient immunodeficient period during adaptive immune maturation offers critical insights into cancer cell differentiation, proliferation, migration, and therapeutic response^83^, ^85^. Therefore, this model holds significant potential for future EBV research, offering a versatile platform to investigate mechanisms of viral oncogenesis and immune interactions that could complement existing approaches. However, the absence of adaptive immunity in early developmental stages restricts its investigations into T cell‐ or antibody‐mediated antiviral responses. Additionally, EBV cannot naturally infect zebrafish, necessitating xenograft approaches that lack viral replication or latency dynamics.

## APPLICATIONS OF EXPERIMENTAL MODELS FOR EBV RESEARCH

### Investigations of the EBV infection mechanism and the viral life cycle regulation

Experimental models are indispensable for elucidating the infection mechanisms and life cycle regulation of EBV. The infection mechanism of EBV involves multiple cell types and complex molecular interactions. Early breakthroughs in understanding EBV entry mechanisms were achieved through pioneering *in vitro* infection models. In 1987, Nemerow et al. first demonstrated the critical role of gp350 in B cell infection and immortalization using primary B lymphocyte infection models^86^. Subsequent studies by Tanner, Ogembo, and colleagues identified CD21/CD35 as the principal receptor complex mediating EBV entry into B cells^87^, ^88^, ^89^. Interestingly, Janz et al. later revealed through epithelial cell infection models that gp350 is dispensable for epithelial cell infection^90^, highlighting cell type‐specific entry mechanisms. In epithelial cells, researchers have demonstrated that EBV entry depends on integrin molecules^91^, ^92^, neuropilin‐1 (NRP1)^93^, and non‐muscle myosin heavy chain IIA (NMHC‐IIA)^94^, as well as the participation of molecules including Ephrin receptors (such as EphA2)^95^ and desmocollin‐2 (DSC2)^96^, ^97^. Importantly, R9AP has been identified as a crucial common receptor of B cells and epithelial cells via its interaction with the viral gH/gL complex^98^. These newly identified receptors allow researchers to create humanized EBV‐susceptible animal models by imparting infection susceptibility to resistant cells, thus furnishing critical targets for vaccine and therapeutic development. *In vitro* epithelial cell or B cell infection models are crucial for studying the molecular mechanisms of the initial recognition, attachment, and internalization between EBV and target cells. Additionally, gene‐editing techniques such as CRISPR/Cas9 are widely used to investigate the function of specific genes during EBV infection via *in vitro* models^99^, ^100^. For instance, Kanda et al. combined CRISPR/Cas9 and PacBio sequencing to characterize EBV genomes from gastric cancer cell lines (SNU719 and YCCEL1), demonstrating that reconstituted cancer‐derived EBVs confer resistance to oncogene‐induced cell death in epithelial cells, thereby enhancing the understanding of EBV pathogenic subtypes in gastric carcinogenesis^101^.

To simulate the natural process of EBV transmission in humans through saliva (including oropharyngeal epithelial cells and B cells) and to study the dynamic processes of virus replication, transmission, and clearance in the body, animal models provide a more complex and holistic *in vivo* system. For example, Okuno et al. inoculated rabbits with EBV through the oral route to simulate the process of natural primary human infection, setting up a model for the *in vivo* study of the mechanism of viral infection^70^. Although the infection rate of rabbits inoculated orally with EBV was lower than that in those inoculated nasally, which is somewhat different from the situation in humans, this does not diminish the wide application and value of animal models in studying viral infection mechanisms.

*In vitro* systems, particularly EBV‐infected B cell lines (e.g., LCLs, Akata, and Mutu) and epithelial cell cultures, have been instrumental in defining viral latency programs, lytic reactivation triggers, and host–pathogen interactions. Studies utilizing EBV‐positive cell lines at distinct latency stages have identified numerous chemical and biological inducers capable of triggering EBV lytic reactivation. Key agents include histone deacetylase (HDAC) inhibitors^102^, protein kinase C (PKC) agonists^103^, DNA methyltransferase (DNMT) inhibitors^104^, anti‐immunoglobulins^105^, and transforming growth factor‐β (TGF‐β)^106^. Mechanistic investigations have further revealed that these inducers predominantly target the viral immediate‐early (IE) gene promoters (e.g., BZLF1 and BRLF1), modulating epigenetic modifications (e.g., histone acetylation and DNA demethylation) or activating cellular signaling pathways (e.g., MAPK/ERK and NF‐κB). Such studies have been instrumental in delineating the molecular switches governing the transition from viral latency to lytic replication, providing critical insights into both EBV pathogenesis and therapeutic strategies aimed at disrupting viral persistence. These *in vitro* induction models have proven invaluable for delineating the temporal regulation and functional significance of EBV gene expression programs during both latent and lytic phases^107^, ^108^, ^109^, significantly advancing our understanding of the viral life cycle and host–virus interactions. Correspondingly, the 3D culture model also provides a reliable research model for studying the regulatory factors of the EBV life cycle. For example, Tugizov et al. first used organotypic raft cultures to demonstrate that EBV traverses polarized oral epithelial layers via transcytosis, a process dependent on apical–basal transport machinery^110^. Subsequent studies by Caves et al. revealed that ALI‐cultured epithelia support spontaneous EBV lytic reactivation at magnitudes unachievable in monolayer systems, bypassing artificial chemical induction^30^. Notably, Yu et al. established ALI‐grown primary nasopharyngeal epithelia, showing that stratified, differentiated layers exhibit heightened EBV susceptibility compared to undifferentiated basal cells^111^. Similarly, Temple et al. showed that EBV establishes a productive infection in suprabasal epithelial layers, co‐expressing latent and lytic proteins and producing viruses without proliferation^45^. This differentiation‐dependent viral behavior aligns with clinical observations, where EBV reactivation is confined to suprabasal layers in oral hairy leukoplakia^45^. These *in vitro* culture methods not only successfully simulate the physiological characteristics of host cells *in vivo* but also partially replace the application of animal models in related research while meeting safety and ethical requirements.

### Investigations of EBV pathogenic mechanisms and the host's immune response

**Figure 3:**
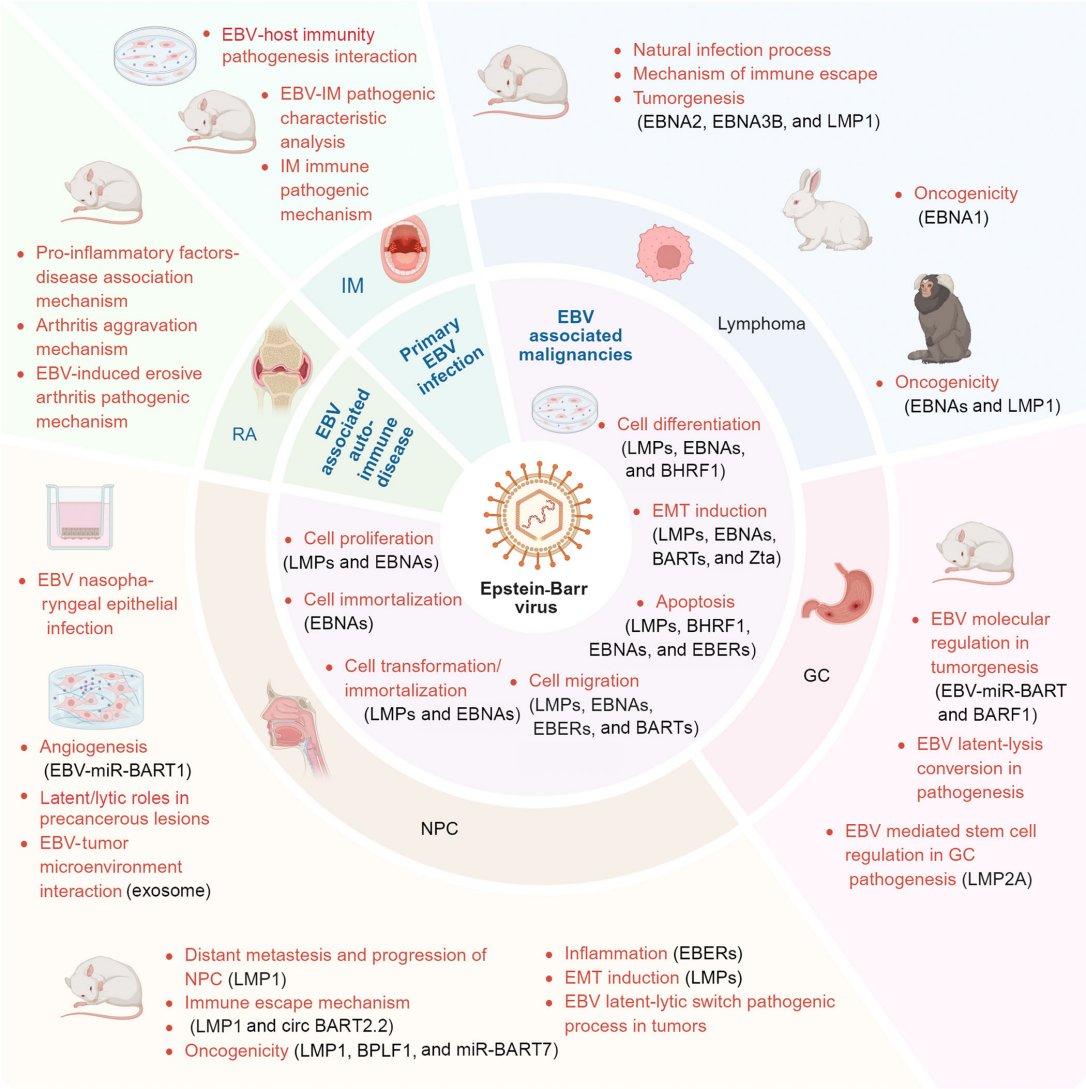
Key pathogenic mechanisms of EBV and associated experimental models. Schematic illustrates how experimental models elucidate the pathogenic contributions of specific EBV genes across associated diseases, including infectious diseases, malignancies, and autoimmune disorders, highlighting the mechanistic insights gained from these experimental platforms. BARTs, BamHI‐A rightward transcripts; BHRF1, BamHI H rightward reading frame 1; EBERs, Epstein–Barr virus‐encoded small RNAs; EBNAs, Epstein–Barr nuclear antigens; EMT, epithelial‐mesenchymal transition; GC, gastric cancer; IM, infectious mononucleosis; LMPs, latent membrane proteins; NPC, nasopharyngeal carcinoma; RA, rheumatoid arthritis; Zta, Z transactivator. This figure was created by Bio‐Render. Wufuer, G. (2026) https://www.biorender.com.

Following EBV infection, host cells initiate a series of complex responses that may lead to varying degrees of pathological changes (Figure 3). At the cellular level, EBV‐induced lesions encompass multiple facets, including apoptosis, cell transformation (such as the immortalization and carcinogenesis of B cells), and abnormalities in the cell cycle and function. EBV precisely regulates apoptosis through its diverse gene products^112^, ^113^. Based on 2D cell lines and mouse models, researchers have revealed that LMP1 can mimic CD40 to activate signaling pathways such as NF‐κB and MAPK to immortalize cells and drive tumor formation^114^. Moreover, these models have provided key insights into the mechanisms by which EBV latent genes and host oncogenes drive cell cycle dysregulation^115^ and genomic instability^116^, thereby accelerating the progression of EBV‐associated tumors. In traditional 2D culture models, immortalized cell lines serve as invaluable experimental platforms for investigating the pathobiological mechanisms underlying EBV‐associated malignancies. These cell lines also provide insights into the fundamental virological characteristics of EBV, including its latency, replication, and transformative potential. Moreover, gastric organoids derived from human induced pluripotent stem cells (iPSCs) have advanced studies on *Helicobacter pylori* but remain underutilized in EBV research^117^.

The integration of *in vitro* models with animal models allows for a more comprehensive and systematic exploration of the pathogenic mechanisms underlying EBV infection. Currently, various animal models have made significant progress in simulating EBV infection‐related diseases. For instance, Smith et al. developed an orthotopic NPC model in severely immunodeficient NOD.Cg‐*Prkdc^scid^Il2rg^tm1Wjl^/SzJ* (NSG) mice, revealing EBV‐encoded BART microRNAs as critical drivers of metastasis^118^, ^119^. Wan et al. generated primary and orthotopic NPC murine models using CRISPR‐engineered 3D spheroids to create a platform for dissecting the carcinogenic functions of EBV in NPC, revealing that *Trp53*, *Cdkn2a*, *TGFBR2,* and EBV‐encoded oncoprotein LMP1 accelerate tumor progression and distal metastasis^120^. Similarly, Murakami et al. demonstrated for the first time that EBV‐positive epithelial cell lines derived from human gastric tissue can form tumors in severe combined immunodeficiency (SCID) mice, providing a valuable model for studying EBV‐associated epithelial malignancies like gastric cancer and NPC^121^. Klaus Rajewsky and colleagues demonstrated that the temporally controlled expression of EBV proteins LMP1 and LMP2A, which mimic constitutively activated CD40 receptor and B‐cell receptor signaling in murine B cells, recapitulates acute EBV infection, providing a preclinical model for B cell lymphomagenesis^122^, ^123^, ^124^. Moreover, proliferative activities of LMP1, LMP2A, and EBNA2 in combination with EBNA3A‐mediated inhibition of terminal plasma cell differentiation critically control EBV‐mediated B cell lymphomagenesis^125^. Additionally, EBV infection can induce rheumatoid arthritis (RA)‐like erosive arthritis or B cell lymphoproliferative diseases in humanized mice, closely mirroring the pathological processes observed in humans following EBV infection^126^. The rabbit model shows splenomegaly and elevated viral loads, features that resemble acute IM, making it valuable for dissecting early immune responses and viral persistence^71^. The tree shrew model can reproduce disease symptoms similar to those of human IM, with EBV infection causing persistent viremia and pathological changes akin to human IM^79^. Huang et al. demonstrated the utility of zebrafish xenografts in modeling NPC, where transplanted human EBV‐positive NPC cells survived, proliferated, and showed extravasation in zebrafish embryos^127^. This model enables real‐time visualization of metastatic behaviors, such as vascular invasion and metastasis in an intact vertebrate system. Their immune‐naïve embryos permit tumor engraftment without host rejection, ideal for studying EBV‐mediated immune evasion^127^. In the study of the pathogenesis of EBV‐related diseases, these *in vivo* and *in vitro* models provide crucial experimental evidence.

The interaction between a virus and its host is a complex and dynamic process, capable of not only stimulating the host's immune response but also prompting infected cells to adopt immune evasion strategies. Currently, most research on the molecular mechanisms of host–EBV interactions and the generation of corresponding immune responses relies heavily on *in vitro* culture models. Additionally, studies on the mechanisms by which EBV achieves immune escape after infecting the host have been conducted using in vitro models. Vilmen et al. demonstrated that the EBV‐encoded BHRF1 protein reduces interferon signaling and promotes immune evasion through mitochondrial division, resulting in the degradation of mitochondrial and mitochondrial antiviral‐signaling (MAVS) proteins, by transfecting relevant genes into cell lines like EBV‐transformed AGS‐Bx1/HEK293T cells^128^. Similarly, Gupta et al. illustrated that the EBV protein BPLF1 achieves immune escape by blocking the interaction between TRIM25 and RIG‐I, thereby inhibiting RIG‐I activation and IFN induction, through *in vitro* transfection of AGS‐Bx1 and HeLa cells^129^.

Regarding the adaptive immune response to EBV infection, Bu et al. demonstrated that the host can produce neutralizing antibodies against gp350, gp42, and gH/gL at different times postinfection by combining *in vitro* and *in vivo* models^130^. Animal models provide a more intricate *in vivo* environment for studying viral infections and are therefore instrumental in investigating the host's immune response. For instance, although humanized immune system mouse strains (NSG, NRG, and BRG) have immune deficiencies and do not reproduce humoral immune responses well, they can still effectively trigger cell‐mediated immune responses during EBV infection. Studies have shown that natural killer (NK) cells, CD4^+^ T cells, and CD8^+^ T cells play a key role in controlling viral infections and related tumor formation^131^. Animal models offer insights that are often unattainable through *in vitro* studies alone by simulating the complex interactions between viruses and the host immune system. These research findings provide a crucial scientific foundation for a comprehensive understanding of virus–host interactions and how hosts combat viral infections through innate and adaptive immune mechanisms.

### Application in prevention and treatment of EBV‐associated diseases

Following primary infection, EBV establishes lifelong latency in B lymphocytes and may reactivate under immunosuppressive conditions, contributing to tumorigenesis. The development of accurate diagnostic methods, effective antiviral therapies targeting both lytic replication and latent infection, and preventive vaccines against primary infection or EBV‐associated malignancies represent critical public health priorities. Both *in vitro* and *in vivo* models play indispensable roles in these research endeavors.

Elevated EBV‐specific antibody titers serve as valuable biomarkers for the diagnosis of EBV‐associated diseases. Early work by Zeng Yi's team in the 1970s utilized B95‐8 cells (for VCA‐IgA) and Raji cells (for EA‐IgA) in immunoenzymatic assays for NPC screening^132^, ^133^, ^134^. With technological advancements, enzyme‐linked immunosorbent assays (ELISAs) and chemiluminescent immunoassays (CLIAs) are now more commonly used. However, the antigens for these assays, especially VCA, are still largely derived from the cell lysates of EBV‐positive cell lines like B95‐8 and Raji^135^, ^136^, ^137^, ^138^, ^139^, ^140^. These antigens remain the standard in large‐scale NPC screening programs^141^, ^142^, ^143^, ^144^. Serological screening has proven to significantly facilitate the early diagnosis of NPC and consequently decrease NPC‐related mortality^142^, ^145^.

Current EBV therapeutics focus on two key strategies: inhibiting lytic replication and eliminating latent reservoirs. Nucleoside analogs, such as acyclic nucleoside phosphonates, have demonstrated efficacy in suppressing EBV DNA replication in chemically induced P3HR‐1 and Akata cell models. However, they are ineffective in clearing latent infections^146^, ^147^. Small‐molecule inhibitors, such as ganciclovir (GCV) or 2,5‐dimethylpyrrolyl benzoic acid, targeting viral proteins (e.g., BALF5 polymerase and BGLF4 kinase) or host factors (e.g., integrins), have also shown promise in 2D cell culture systems^148^, ^149^. Ding and colleagues established NPC PDOs, linking drug response heterogeneity to genomic mutations and proteomic profiles^150^. Wang et al. further optimized NPC organoid culture, creating the first live biobank for high‐throughput drug screening^151^. These models are now recognized by regulatory agencies as viable alternatives for preclinical testing when animal models are inadequate^152^.

For the treatment of EBV‐associated tumors, antiviral agents have been demonstrated to be effective. Cidofovir showed tumor growth inhibition in EBV‐positive NPC CDXs, with enhanced apoptosis when combined with ribonucleotide reductase inhibitors (hydroxyurea and didox)^153^, ^154^, ^155^. This approach also reduced EBV oncoprotein expression and improved radiosensitivity in EBV‐associated BL and NPC^155^. Chemotherapeutics and epigenetic modifiers (e.g., HDAC inhibitors) affecting DNA synthesis and drugs influencing host DNA methylation and histone deacetylation can effectively reactivate EBV from latency, triggering the expression of viral proteins capable of eliciting strong host immune responses and sensitizing tumor cells to antiviral agents. Feng et al. demonstrated this strategy *in vitro* across multiple EBV‐positive cell lines wherein combining HDAC inhibitors with chemotherapeutic drugs achieved significant killing effects on EBV‐positive cells^156^. They further validated the anti‐tumor efficacy of this approach *in vivo* using an LCL xenograft model in SCID mice^156^. Subsequent incorporation of antiviral drugs into this regimen further suppressed tumor progression, and this approach was later translated into clinical trials^157^, ^158^. Additionally, Sugiokto et al. utilized CRISPR‐dCas9‐mediated activation of the EBV immediate‐early gene *BZLF1* in an *in vitro* EBV‐positive tumor cell culture model, which induced lytic replication and enhanced tumor cell killing when combined with GCV^159^. This method, termed cytolytic virus activation (CLVA) therapy^160^, has shown safety and efficacy in phase I/II trials for recurrent NPC^161^. Additionally, zebrafish excel in high‐throughput drug screening due to their rapid development, low cost, and ease of genetic manipulation^162^. A key advantage is the ability to engraft patient‐derived tumors or create transgenic models in large zebrafish cohorts, thereby enabling comprehensive evaluation of numerous clinically available drugs^162^.

The development of effective vaccines against EBV represents a critical strategy for controlling EBV infection and its associated diseases. Both *in vitro* and *in vivo* models are essential for evaluating vaccine efficacy. The glycoprotein gp350 has emerged as a primary candidate due to its abundance on the surface of infected cells and viral particles, as well as its crucial role in mediating EBV infection^90^, ^163^. The protective potential of gp350‐based vaccines was first established in 1985 in cotton‐top tamarins and common marmosets (*Callithrix jacchus*), which elicited robust neutralizing antibody responses and prevented EBV‐induced lymphomagenesis^164^. Subsequent advances included recombinant viral vectors (adenovirus and vaccinia) and nanoparticle formulations, which effectively protected cotton‐top tamarins or common marmosets from lymphoma development and lethal EBV infection^165^, ^166^, ^167^, ^168^, ^169^. Subsequent research has expanded the repertoire of EBV vaccine candidates to include other viral antigens such as EBNA1, gH/gL, LMP2, and gB. These developments have relied heavily on *in vitro* neutralization assays using cell culture systems, complemented by challenge studies in humanized mouse models that provide critical assessment of protective efficacy^170^, ^171^, ^172^, ^173^, ^174^, ^175^.

## CONCLUDING REMARKS

*In vitro* and *in vivo* models of EBV‐associated diseases have significantly advanced our understanding of viral pathogenesis, host–pathogen interactions, and therapeutic development. *In vitro* systems, such as immortalized B cell lines and organoids, offer unparalleled simplicity, cost‐effectiveness, and scalability for high‐throughput drug screening and mechanistic studies of viral latency and lytic reactivation. However, their inability to recapitulate the dynamic interplay between EBV and the human immune system, as well as tissue‐specific drug metabolism, limits translational relevance. Conversely, *in vivo* models, including humanized mice, rabbits, tree shrews, and zebrafish xenografts, bridge this gap by preserving physiological complexity, enabling studies of immune evasion, metastasis, and therapeutic biodistribution. Nevertheless, interspecies discrepancies in immune gene networks, ethical constraints, and incomplete viral tropism underscore persistent challenges in achieving clinical fidelity. The advantages and disadvantages of different models are summarized in Table 1.

**Table 1:** Comparison of Epstein–Barr virus (EBV) experimental models.

Classification	Model	Complexity	Time	Cost	Micro‐environment simulation	Immune system	Application
*In vitro* model	2D cell line	Low	Hours to days	Low	Poor	None	Research of EBV infection and pathogenic mechanisms; viral–host interaction; drug efficacy evaluation
	3D culture	Moderate–high	Weeks	Moderate	Limited	Partial	
Animal model	Non‐human primate	High	Weeks to months	Very high	Effective	Full	Drug/vaccine efficacy evaluation; research of EBV infection and pathogenic mechanisms
	Rodent	High		High		Reduced/Partial	Drug/vaccine efficacy evaluation; research of EBV infection and pathogenic mechanisms; EBV‐driven carcinogenesis
	Rabbit	High		High		Full	Research of EBV infection
	Others	High		High		Full	EBV‐driven carcinogenesis

Complexity and cost levels are categorized as follows: “Low” refers to simplified systems with limited physiological interactions and minimal requirements for reagents and maintenance; “High” refers to advanced models that closely mimic the human tumor microenvironment or immune system, necessitating specialized animal housing, long‐term husbandry, and ethical compliance.

Recent technological innovations, such as CRISPR‐engineered organoids, 3D bioprinted tissue constructs, and multi‐omics integration, are reshaping EBV research paradigms. Organoid models incorporating stromal and immune components now partially emulate the nasopharyngeal TME, while humanized mice with reconstituted adaptive immunity better mimic EBV‐driven lymphomagenesis. Furthermore, single‐cell RNA sequencing has identified novel EBV–host interactomes in these models. Despite these advances, critical knowledge gaps remain, particularly in modeling lifelong EBV persistence, intermittent reactivation, and tissue‐specific oncogenesis.

Future research should prioritize the development of high‐fidelity hybrid models that synergize *in vitro* precision with *in vivo* physiological contexts. Strategies include (1) engineering “humanized” animal models with CRISPR‐inserted EBV entry receptors to enable natural infection; (2) co‐culturing PDOs with autologous immune cells to study personalized antiviral responses; and (3) leveraging machine learning to predict EBV oncoprotein–host protein interactions from multi‐omics data, accelerating drug updating and iteration. Concurrently, ethical frameworks for advanced *in vivo* models must evolve to balance scientific rigor with animal welfare. By integrating these approaches, next‐generation models will unravel EBV's lifelong pathogenesis and catalyze curative therapies for its associated malignancies.
